# Breast cancer specialists’ experiences and attitudes towards mainstream genetic testing for patients with breast cancer

**DOI:** 10.1186/s13053-026-00340-3

**Published:** 2026-04-24

**Authors:** Kirsten Allan, Linda Cicciarelli, Catherine Beard, Geoffrey J. Lindeman, G. Bruce Mann, Paul A. James, Laura E. Forrest

**Affiliations:** 1 Department of Paediatrics, The University of Melbourne, Parkville, 3010 Australia; 2 Parkville Familial Cancer Centre, Peter MacCallum Cancer Centre, Melbourne, Victoria 3000 Australia; 3 Department of Medicine, The University of Melbourne, Parkville, 3010 Australia; 4 The Walter and Eliza Hall Institute of Medical Research, Melbourne, Australia; 5 Department of Surgery, The University of Melbourne, Parkville, 3010 Australia; 6 Sir Peter MacCallum, Department of Oncology, The University of Melbourne, Parkville, 3010 Australia

**Keywords:** Mainstreaming, Breast cancer, Genetic testing, Oncology professionals, Oncology, Attitudes

## Abstract

**Background:**

Germline genetic testing is an increasingly important component of treatment decision-making for clinicians and patients with breast cancer. To address increased demand and expedite access to genetic testing for these patients, the Parkville Familial Cancer Centre (PFCC) in Victoria, Australia, implemented a breast mainstream genetic testing program. The program educates and supports breast cancer specialists to provide eligible patients with pre-test information, gain consent, and arrange genetic testing during their routine cancer appointments. This study aimed to explore breast cancer specialists’ experiences and opinions of the education program and of facilitating mainstream genetic testing for their patients.

**Methods:**

Specialists who had attended the mainstream genetic testing education were invited to complete an online survey about the training provided through the education program and their experience of deploying mainstream genetic testing in their practice. Descriptive statistics were compiled, and content analysis used for open text responses.

**Results:**

Forty-five breast cancer specialists (breast surgeons, medical oncologists, radiation oncologists and breast care nurses) responded (45% response rate). Most respondents had discussed (87%) and consented (80%) patients for mainstream genetic testing. Most specialists (81%) rated their confidence levels as high or very high for consenting patients to mainstream genetic testing. The majority (89%) indicated that they believed mainstream genetic testing should be part of their role and felt well supported by the PFCC (90%).

**Conclusion:**

Breast cancer specialists used the education they received in the mainstream education program and were supported to deliver mainstream breast cancer genetic testing to their patients.

**Supplementary information:**

The online version contains supplementary material available at 10.1186/s13053-026-00340-3.

## Introduction

The significance of a germline pathogenic variant to inform treatment and surgical decision-making for patients with breast cancer has come to the forefront in recent years [1–5]. Genetic information has a critical role in breast cancer treatment, management, and subsequent risk reduction. This role is exemplified through the use of PARP inhibitors in *BRCA*-associated cancer [3, 6], avoidance of radiotherapy in individuals with a germline *TP53* pathogenic variant [1], and guidance regarding the extent of surgical intervention including mastectomy rather than breast conservation, contralateral risk reducing mastectomy and bilateral salpingo-oophorectomy offered as risk reducing strategies, and the latter also an adjunct for endocrine therapy [5]. Given that the efficacy of these novel breast cancer treatments depend on an individual’s germline pathogenic variant status, it is important that patients who meet criteria where the probability of identifying a pathogenic variant is 10% or higher can access genetic testing efficiently [7].

Traditionally, patients with breast cancer have accessed genetic testing via a referral to a clinical genetics service as only medical specialists employed by these services could order genetic testing. This model sees patients attend two appointments pre- and post-genetic testing where they receive genetic counselling provided by genetic health professionals to support their decision-making about the testing, and disclosure of results with counselling about the personal and familial implications. However, increased demand and consequent waiting times for genetics appointments have detrimentally impacted timely access for patients with breast cancer. New models of care have been devised to facilitate access to patients whose care hinges upon the identification of germline information [8–10].

The ‘mainstream’ model, whereby a non-genetic specialist working externally to a clinical genetics service facilitates patient consent and requests genetic testing, has revolutionised access to genetic testing and has subsequently expedited this process for patients. Internationally, mainstreaming models were first deployed for ovarian cancer genetic testing [8, 11], followed by breast [12–15], and more recently prostate and endometrial cancer [16, 17]. Patient outcomes have uniformly demonstrated satisfaction, low decisional regret and post-test distress [16]. In concert with the delivery of mainstream genetic testing, educational programs have been developed to upskill non-genetic specialists and increase understanding of how to facilitate genetic testing and disclose genetic test results [18, 19]. Despite the existence of these mainstream education programs, internationally, a lack of training opportunities for clinicians delivering mainstream genetic testing has been identified [20].

**Fig. 1:**
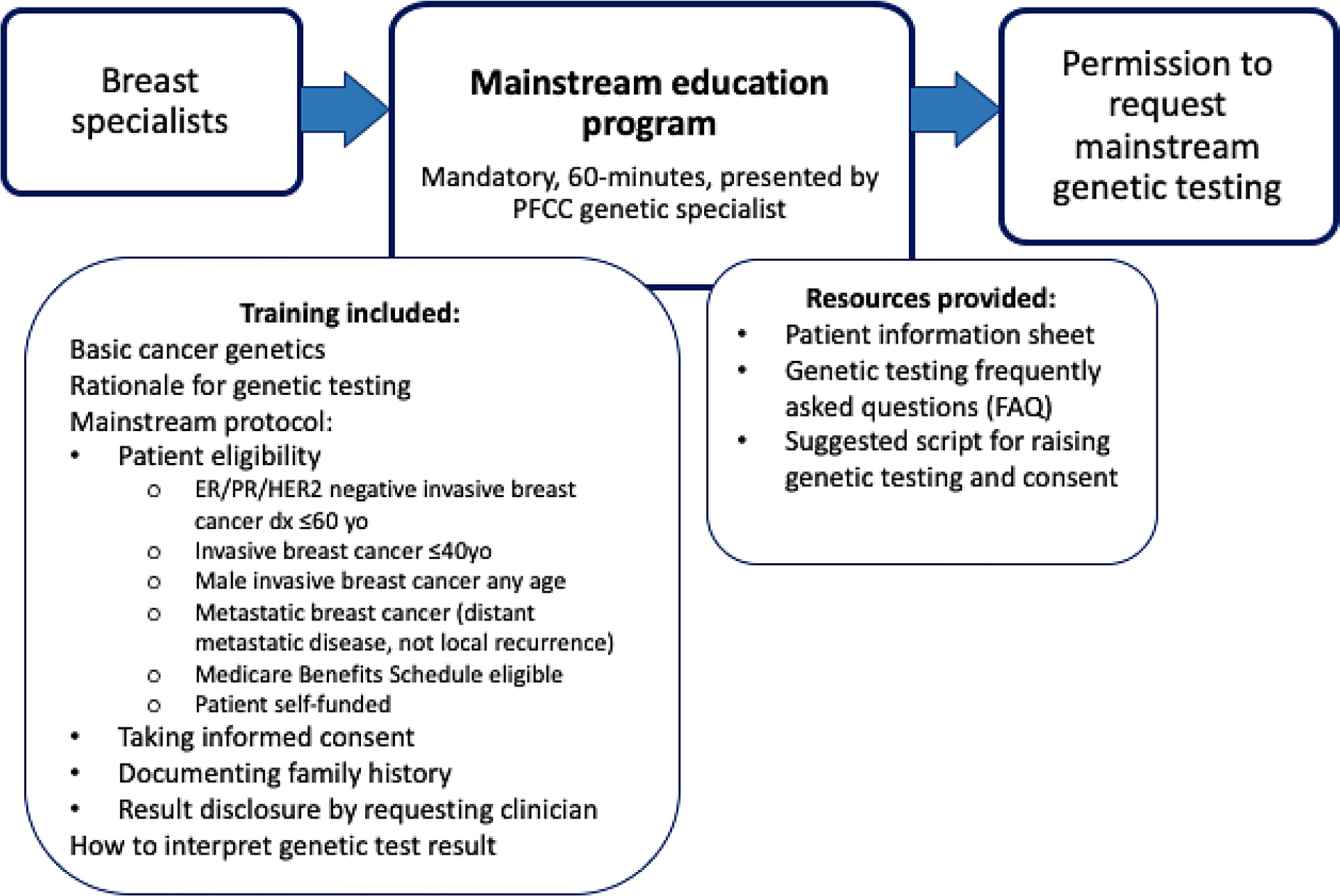
PFCC mainstream education program

The Parkville Familial Cancer Centre (PFCC) in Melbourne, Australia, established a breast cancer mainstream genetic testing program in 2017 [15]. This program begins with an educational program for breast cancer specialists (see Fig. 1) and then supports the specialists to facilitate mainstreaming at ten hospitals in the State of Victoria, Australia. In the PFCC model, the breast cancer specialists organise genetic testing during an oncology, surgery, or radiation appointment and follow up results as part of the patients’ routine clinical care. The PFCC oversees all genetic tests ordered and the laboratory informs both the breast specialist and the PFCC of the test results when available. The treating breast cancer specialist is subsequently responsible for any treatment implications, while the PFCC provides a letter outlining test results to all patients. For patients who receive positive results additional genetic counselling and a confirmatory genetic test is provided by the PFCC, as per standard clinical genetic test requirements. Breast cancer patients with uninformative results and a significant family history of cancer are also reviewed by the PFCC and offered genetic counselling about residual cancer risks, cancer risk management, familial implications and further genetic investigations, if indicated [15]. Given this change in the role of the breast specialist in facilitating patients’ access to genetic testing, it is important to understand whether the non-genetic specialists are being appropriately educated and trained to facilitate mainstream genetic testing and their experiences of facilitating this model of care. Therefore, the aim of this study is to examine the breast cancer specialists’ opinions of the training they received via the mainstream education program, their experiences of facilitating mainstream genetic testing for their patients, and their attitudes towards the mainstream model of care.

## Methods

### Research design

A cross-sectional study was designed using an online survey with purpose-designed questions to examine breast cancer specialists’ experiences of the mainstream education program and facilitating mainstream genetic testing.

### Ethics

Ethics was approved by the Peter MacCallum Cancer Centre Human Research Ethics Committee (HREC LNR/52472/PMCC-2019, Peter Mac Project No: 19/57L).

### Participants

All breast cancer specialists (medical oncologists, radiation oncologists, breast surgeons and breast care nurses, herein referred to as ‘breast cancer specialists’) who had attended a PFCC breast cancer mainstream education program between 2017 and 2019 were eligible to participate. Overall, a total of 108 breast cancer specialists participated in the breast mainstream education program by June 2019, at ten sites across Victoria, Australia: Peter MacCallum Cancer Centre, The Royal Melbourne Hospital, The Royal Women’s Hospital, Western Health, St Vincent’s Hospital, Box Hill Hospital, Bendigo Health, South West Healthcare (Warrnambool), and the Andrew Love Cancer Centre Geelong. Five breast cancer specialists could not be contacted and therefore were excluded from the study. One hundred and three breast cancer specialists were eligible for recruitment and included: 55 medical oncologists, 10 radiation oncologists, 32 surgeons and 6 breast care nurses.

### Survey design

Survey domains included demographic information (6 items), experience of the mainstream education program (5 items), experience of implementing mainstream genetic testing into their practice (17 items), and attitudes towards mainstream genetic testing (6 items). The survey primarily used close-ended questions with some open-ended questions. The survey was hosted online using REDCap at the Peter MacCallum Cancer Centre (see Supplementary material 1) [21, 22].

### Data collection

Data were collected from 24th June 2019 until 30th March 2020. Participants were invited via email which included a link to the online participant information, consent page, and survey. Progression through the survey was dependent on respondents’ clinical experiences of mainstream genetic testing and the number of participants who completed each question is reported.

### Data analysis

Data were analysed using STATA Data Analysis and Statistical Software version 14 (StataCorp, Texas). All responses were summarised using numbers and percentages, and continuous data were analysed to produce means and standard errors of the mean. Content analysis was used where a member of the research team (author: KA) inductively coded the open text responses to identify meaningful content and group common responses.

## Results

Forty-nine breast cancer specialists responded to the survey (48% response rate), with 45 responses included in the analysis. More responders had ordered a mainstream genetic test than the non-responders (*p* = 0.049), however, there was no difference observed in terms of the average number of mainstream tests ordered per group (Table 1). Almost 70% of respondents had ordered a mainstream genetic test, and had ordered 9 tests on average.

**Table 1:** Responders compared to non-responders’ mainstream genetic test delivery

	Responders *N* (%)	Non-responders *N* (%)	Total *N* (%)	*P* value
*N* (%)	49 (47.6)	54 (52.4)	103 (100)	
Ordered mainstream genetic testing (Y)	33 (67)	26 (48)	59 (57)	0.049^a^
Number of mainstream tests ordered				
Mean (SD)	9.1 (11.1)	5.0 (5.1)	7.3 (9.2)	0.088^b^
95% CI	5.1, 13.0	2.9, 7.0	4.9, 9.6	

a. Pearson chi^2^=3.90

b. t=-1.74, df = 57

Most of the respondents worked in the public health sector and in metropolitan services (Table 2). 78% of respondents reported they had not received any formal training in genetics preceding the mainstream genetic testing program.

**Table 2:** Participant demographics

Category	Medical Oncologist (*n* = 22)	Radiation Oncologist (*n* = 3)	Surgeon (*n* = 16)	Breast Care Nurse (*n* = 4)	Total (*n* = 45)
Sex					
Female	13 (59%)	2 (67%)	9 (56%)	4 (100%)	28 (62%)
Male	9 (41%)	1 (33%)	6 (38%)	0 (0%)	16 (36%)
Prefer not to answer	0 (0%)	0 (0%)	1 (6%)	0 (0%)	1 (2%)
Years in profession					
< 10 years	13 (59%)	1 (33%)	6 (38%)	1 (25%)	21 (47%)
≥ 10 years	9 (41%)	2 (67%)	10 (62%)	3 (75%)	24 (53%)
Professional sector					
Public	20 (91%)	3 (100%)	14 (87%)	4 (100%)	41 (91%)
Private	2 (9%)	0 (0%)	2 (13%)	0 (0%)	4 (9%)
Location of practice					
Metro	15 (68%)	3 (100%)	15 (94%)	2 (50%)	35 (78%)
Rural	6 (27%)	0 (0%)	1 (6%)	2 (50%)	9 (20%)
Both	1 (5%)	0 (0%)	0 (0%)	0 (0%)	1 (2%)

### Experiences of the mainstreaming education program

87% (*n* = 39/45) of breast cancer specialists reported the education program provided them with the skills to discuss mainstream genetic testing and 76% (*n* = 34/45) reported that they gained the skills to consent patients for mainstream genetic testing. All four nursing respondents indicated that the education program did not provide them with the skills to consent patients for mainstream genetic testing but clarified with open text responses that this was because consenting patients for testing was not within their professional role.

64% (*n* = 29/45) of breast cancer specialists reported that the education program provided them with the skills to interpret mainstream genetic testing results for patients. Of those who did not feel they gained the skills to interpret a result, four of eight respondents explained using open-text that interpreting and explaining a variant of uncertain significance remained challenging after the education program.

Overall, breast cancer specialists agreed that the education program had clear objectives (*n* = 37/45; 82%), was relevant to their practice (*n* = 37/45; 82%), was well organised (*n* = 38/45; 84%), easy to follow (*n* = 36/45; 80%), and the one hour allocated to the program was sufficient (*n* = 36/45; 80%).

### Experiences of facilitating mainstream genetic testing

**Fig. 2:**
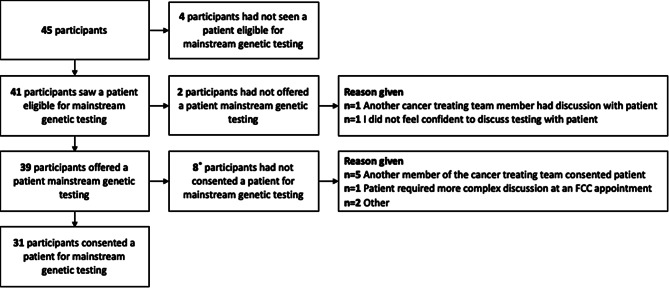
Flow chart of participants’ clinical experience of offering mainstream breast cancer genetic testing. *includes four breast care nurses who were not eligible to consent patients in the PFCC program

A flow chart of the study participants’ clinical experience of the mainstream program is shown in Fig. 2. 91% (41/45) of breast cancer specialists had seen an eligible patient since completing the education program. Of those 41 participants, 39 had raised the option of mainstream genetic testing with a patient. Thirty-one participants (*n* = 31/39) had consented at least one patient for mainstream genetic testing.

18% (7/39) of participants identified that they had seen an eligible patient but did *not* offer them mainstream genetic testing. Four of those seven participants indicated that the reason for not offering testing to an eligible patient was that the “patient required a more complex discussion at an FCC appointment”, while the remaining three participants stated they “did not have the time required to discuss testing” with the patient.

Participants were asked to report the number of patients they had offered mainstream genetic testing to and/or had consented for mainstream genetic testing. All professional groups reported similar average numbers of patients who they had discussed mainstream genetic testing with since the program’s implementation. This was also true for the average number of patients who radiation oncologists (5, *n* = 1), medical oncologists (5 ± 0.6 SEM, *n* = 18) and surgeons (6 ± 1.3 SEM, *n* = 12) had consented for mainstream genetic testing.

80% of participants reported having high (*n* = 18/31, 58%) or very high (*n* = 7/31, 23%) confidence levels when consenting patients for mainstream genetic testing. The frequency in which participants mentioned 13 key points to discuss during consent process is detailed in Table 3.

**Table 3:** Reported frequency that key points were discussed by breast cancer specialists’ during the consent process

When consenting a patient for mainstream genetic testing, do you discuss the following: (*n* = 31)	Never or Rarely *n* (%)	Sometimes *n* (%)	Always *n* (%)
How the sample will be collected	1 (3)	3 (10)	27 (87)
The cost involved	3 (10)	9 (29)	19 (61)
That the results will be used for clinical and not research purposes	7 (23)	10 (32)	14 (45)
The mode of inheritance of hereditary breast cancer genes	4 (13)	11 (35)	16 (52)
Possible family implications	0 (0)	3 (10)	28 (90)
Privacy concerns	3 (10)	14 (45)	14 (45)
Possible insurance implications	1 (3)	8 (26)	22 (71)
The expected time until results are available	0 (0)	4 (13)	27 (87)
The types of results (no mutation, mutation, variant of unknown significance)*	0 (0)	7 (23)	24 (77)
What the different types of results mean in terms of the risk of developing another cancer	3 (10)	13 (42)	15 (48)
What the different types of results may mean for current treatment plan	1 (3)	6 (20)	24 (77)
Provide patient with a copy of the “Patient Information Sheet”	1 (3)	8 (26)	22 (71)
Discuss referring to FCC for further counselling/support	2 (6)	7 (23)	22 (71)

*Terminology used during 2017

All participants (*n* = 29/29) personally ordered and signed the pathology request slip for their patient once they had consented to mainstream genetic testing. 97% of participants (*n* = 28/29) always (*n* = 15/29) or sometimes (*n* = 13/29) disclosed the mainstream genetic testing results directly to their patients. Of those who did not ‘always’ deliver the results to their patient, 57% (n = 8/14) nominated that the majority of the time the PFCC disclosed the mainstream genetic testing result. 66% of participants (n = 19/29) had at least one patient who had received a positive mainstream genetic testing result. Of these 19 participants, 16 indicated that the positive result altered their patient’s breast cancer treatment plan.

### Opinions of mainstream genetic testing

89% of participants (*n* = 40/45) believe that breast mainstream genetic testing should be part of their practice. 90% of breast cancer specialists (*n* = 26/29) felt they had been well supported by the PFCC mainstream program. 80% of breast cancer specialists (*n* = 31/39) did not believe that there was one medical specialist with cancer genetics experience in their clinic who saw a majority of the patients who were eligible for mainstream genetic testing.

Participants indicated that the greatest advantage of mainstream genetic testing was the streamlined process and the rapid turnaround time (see Table 4). Notably, 40% of participants perceived “no disadvantages” of the mainstream genetic testing program.

**Table 4:** Breast cancer specialists’ perceived advantages and disadvantages of the mainstream genetic testing program (*n* = 30)

**Advantages**	**n (%)**
Streamlined process	27 (90)
Rapid turnaround time	26 (87)
Working in direct partnership with PFCC	23 (77)
No advantages	0 (0)
**Disadvantages**	**n (%)**
Increased time involved compared to standard care	13 (43)
Increased management of patient’s stress/anxiety	2 (7)
Increased pressure to provide ‘genetic counselling’	7 (23)
The increased uncertainty for patient	2 (7)
No disadvantages	12 (40)

In the open text comments at the conclusion of the survey, 46% of participants (*n* = 19/41) took the opportunity to suggest improvements to the mainstream genetic testing program or provided comments. Three areas were identified, (1) improvements in communication to oncology teams, (2) improvements in mainstream genetic testing processes, and (3) clarification of the mainstream genetic testing processes (see Table 5). A high level of praise for the mainstream genetic testing program was also identified. A medical oncologist wrote, “*very happy with the program. Glad to be able to offer testing to my patients”*.

**Table 5:** Areas of improvement for the mainstream program suggested by respondents

Common areas for suggested improvements of mainstream genetic testing program	Example of response
Improvements in communication to oncology teams	Surgeon #2: “Communication e.g., email, to tell us (requesting doctor) that a sample has been collected and genetic testing is occurring and the expected date of the result.”
Improvements in mainstream genetic testing processes	Surgeon #10: “Medicare funding only offered for one set of testing. Therefore I’m reticent to muck up initial panel.”
Clarification of the mainstream genetic testing processes	Surgeon #7: “I am not clear whether the FCC will be involved in counselling patients about their results, whether positive or non-informative. Will they only be involved with a positive result?”

## Discussion

This study examined the PFCC breast cancer mainstream program from the perspective of the non-genetic breast cancer specialists who had received education to deliver mainstream genetic testing. Overall, the breast cancer mainstream program was positively received by the breast cancer specialists, which suggests a successful integration of genetic testing into routine breast oncology care in Victoria, Australia. Other clinical services that have implemented mainstreaming programs for genetic testing for breast cancer [12, 14, 23] and ovarian cancer [8, 9, 24], have also reported a high level of clinician support and satisfaction.

### Mainstreaming education program

Breast cancer specialists attended a mandatory education program to upskill in the facilitation of genetic testing for their patients with breast cancer through the PFCC mainstream program. The majority of participants in this study held positive views of the breast mainstream education program and reported that the training provided them with the skills to discuss (87%), consent (76%), and interpret a genetic test result (64%). Gleeson et al. (2020) reported a similar training method used to upskill Australasian non-genetic cancer specialists to offer *BRCA1/2* testing to women with ovarian cancer [18]. This training method was successful in increasing clinicians’ genetic knowledge, communication skills, and willingness to embrace mainstreaming [18]. However, with only 58% of cancer specialists subsequently ordering genetic testing it is notable that education alone does not address system-level barriers [18]. This evaluation study has demonstrated the value in including both education and support for clinicians in implementing mainstream genetic testing, with clinicians responding favourably to the education and 95% of respondents offering mainstream genetic testing to their patients.

Concerns about new oncology staff commencing work at the mainstream hospital sites, who had not attended the education program, was raised by the PFCC program [15]. This suggests that an annual up-skilling education program or uploading recorded sessions online may be necessary to ensure all practicing clinicians have attended appropriate training [15, 19]. Similar mainstreaming programs in the United Kingdom and France have utilised pre-recorded on-line training modules to upskill non-genetic cancer specialists [8, 23, 24]. Clinicians involved in each program reported a high level of satisfaction with the flexible training method, which was accessible at their own convenience and felt well-informed by the training videos [23, 24]. In the future, perhaps using a combination of face-to-face education with online training modules will ensure that breast cancer specialists are regularly up skilled at the PFCC.

Previous studies have reported cancer clinicians have insufficient genetics knowledge and expressed educational needs when it comes to discussing cancer genetics within their consults [24]. Whilst some breast cancer specialists reported concern around their ability to interpret test results that included variants of uncertain significance (VUS) after attending the education program, the PFCC refined the program after rollout to ensure more support around interpretation was offered to mainstream clinicians when a VUS result was reported [15]. The majority of breast cancer specialists in this study self-assessed their confidence levels for consenting patients for mainstream genetic testing as high or above. This finding is consistent with other studies from similar mainstreaming programs [9, 14, 19, 24, 25]. The confidence did not seem to be dependent on the breast cancer specialists’ previous genetics training, as 78% of the participants reportedly had no formal genetics training preceding the mainstream genetic testing program. It is possible that the specialists’ confidence to consent patients could be attributed to the education program that they received.

In essence, the reported overall positive experiences suggests that the training method utilised in the PFCC mainstream program is acceptable and sufficient to support non-genetic breast cancer specialists to incorporate mainstream genetic testing into their practices. Nevertheless, the suggestions for improvement to the PFCC mainstream program offered by the breast specialists could be incorporated into the training program. Specifically, clarifying and delineating the distinct roles of the PFCC as a clinical genetics service and that of the molecular genetics laboratory could encourage breast specialists to contact the laboratory directly to determine turnaround times for test results and encourage a relationship directly between these service providers.

### Clinical experience

Previous mainstream studies suggest that having ‘mainstream champions’ can be a key factor in ensuring mainstream program success. Mainstream champions are non-genetics health professionals practicing in the oncology setting who advocate for genetic testing, and so can facilitate and support the change to practice within the oncology team [15, 26, 27]. The PFCC operates within a tertiary referral centre and has multiple breast specialists who have dual roles within both oncology teams and the PFCC, who have been acting in mainstream champion roles since the introduction of the program. This is supported by participants’ comments in the open-text responses suggesting that there are strong advocates for breast mainstream genetic testing associated with the PFCC mainstream program, ultimately resulting in the program’s successful integration into standard care pathways.

To our knowledge this is the first study that has investigated how frequently key information is discussed during the mainstreaming consent process, as reported by the clinicians. Reassuringly many of the key points, such as treatment implications, family implications and insurance implications were reportedly ‘always’ discussed by 70% or more of the breast cancer specialists when consenting their patients for mainstream genetic testing. A study by Colombo and colleagues evaluated the patient experience of a streamlined oncology-led *BRCA1/*2 testing pathway in America and Europe [24]. Patients assessed the ‘quality’ of pre-test counselling they received from the oncology team, by reporting if their oncologist or nurse discussed 13 elements during the consent process [24]. 99% of patients were satisfied with the pre-test counselling, with over 90% of patients reporting that each of the 13 elements were discussed. The discussions in a mainstreaming context may not be as comprehensive as during a genetics consultation, but evidence from previous studies shows that this is adequate and preferential to patients during a treatment-focused test [28–30]. Importantly in the current study, 94% of breast cancer specialists discussed referring their patient to a genetic service if they wished for a more detailed discussion or psychosocial support. Initial reports from the PFCC mainstreaming program indicate that patient satisfaction is also high [15], however, a more comprehensive study is warranted to fully gauge the patient experience.

### Opinions of mainstream genetic testing

Most breast cancer specialists (89%) believed that mainstream genetic testing should be part of their practice, which demonstrates strong support for the PFCC mainstream program. The identified advantages and challenges of the PFCC mainstream program in this study, closely aligned with previously published studies [8, 9, 13, 31]. Participants nominated several advantages of the breast mainstream program, which included, the streamlined process for patients to access genetic testing, rapid results and collaborating professionally with the genetic service.

The major disadvantage of breast mainstream genetic testing identified in this study was the increase in workload for breast cancer specialists as compared with standard care. Several studies also identified the increased time as a disadvantage of mainstreaming [9, 32–34]. An alternative model adopted in Ireland deployed advanced nurse practitioners, rather than oncologists due to workforce capacity limitations, to facilitate mainstream genetic testing [35]. While this program proved to be feasible, efficient, and effective, genetic testing in Australia must be ordered by a medical practitioner to be publicly funded via the Medical Benefits Schedule. This limits the scope of practice for genetic counsellors and nurses in terms of requesting genetic testing. While the time required for genetic testing was identified as a disadvantage by the breast cancer specialists in this study, they were not deterred, with most reporting that they believed mainstreaming should be part of their role. As breast cancer specialists’ experience and confidence increases, the time required to discuss genetic information and testing in standard cancer care may diminish with more streamlined conversations [36].

### Study limitations

A limitation of this study was that experience of the mainstream program including confidence levels, how many patients the clinicians had consented to testing and what was included in the genetic testing consent discussion was all self-reported, and unable to be verified. This study may also be limited by a response bias, which could influence the results. The breast cancer specialists who have had positive experiences implementing mainstream genetic testing into their practice and who perceived mainstream genetic testing to be important, may have been more likely to respond to the survey. This may have resulted in a more positive representation than the true experience among all breast cancer specialists. The study response rate of 48% of individuals who had committed to attending and completing the training may support this bias and for these reasons, the opinions and attitudes voiced in this study may not be generalizable to the wider community of cancer specialists.

## Conclusion

The data generated from this study suggests that the PFCC has successfully integrated mainstream genetic testing for patients with breast cancer into multiple hospital sites in Victoria. The breast cancer specialists were highly supportive of this model of care, satisfied with the training they received, successfully facilitated testing for their patients and felt positively about integrating testing into their practices. Further improvements with communication between the genetics laboratory and oncology teams will enhance the program’s future progress. Investigation into the patient experience will be a necessary step in the evaluation of the PFCC mainstream program.

## Supplementary Information

Below is the link to the electronic supplementary material.

Supplementary Material 1

## Data Availability

Data generated by this survey research that supports the findings of this study are not available due to the risk of identifying participants from their responses.
