# Supplementary material for: Breast cancer specialists’ experiences and attitudes towards mainstream genetic testing for patients with breast cancer
Source: Hered Cancer Clin Pract. 2026 Apr 24;24:14. doi: 10.1186/s13053-026-00340-3 (PMC13262353; doi:10.1186/s13053-026-00340-3)
Supplement: Supplementary file 1 — Supplementary Material 1 [file 13053_2026_340_MOESM1_ESM.pdf]

Exploring cancer specialists' experiences and attitudes towards facilitating mainstream genetic testing for patients with breast cancer.

Information and consent to participate

You have been invited to participate in a study being conducted by the Parkville Familial Cancer Centre at the Peter MacCallum Cancer Centre. The aim of this project is to evaluate the ‘Breast Mainstreaming’ model of care. We are asking cancer specialists who have attended a ‘Breast Mainstreaming’ in-service, to help us evaluate this training method and explore their personal experiences and views towards offering genetic testing to their patients.

We hope it will help us to improve our mainstreaming model of care across oncology services in Victoria.

As a specialist that has attended this training, we value your opinion of the in-service as well as how you have personally experienced offering patients mainstream genetic testing in your practice.

What will I be asked to do?

Should you agree to paticipate, you will be asked to complete a survey. The survey has 4 sections, which will take approximately 20 minutes of your time.

If you commence the survey, this means you are providing consent to participate in this research study.

The information will be anonymous and confidential and stored securely in the Parkville Familial Cancer Centre at the Peter MacCallum Cancer Centre. Only the researchers involved with this project will have access to this information.

At the end of the project, results in de-identified and aggregate form may be presented at conferences or published in medical journals. We are required to keep information collected as part of this research for at least 5 years following the last publication of the project.

Where can I get further information?

Should you require any further information, or have any concerns about the content of this survey, please do not hesitate to contact:

Genetic Counsellor: Ms Linda Cicciarelli

Email: linda.cicciarelli@petermac.org

Telephone: 03 8559 5322.

Master of Genetic Counselling student: Dr Kirsten Allan

Email: k.allan1@student.unimelb.edu.au

Should you have any concerns about the conduct of the project, you are welcome to contact the Ethics Coordinator, Human Research Ethics, Peter MacCallum Cancer Centre (P: 03 8559 7540 | E: ethics@petermac.org)

This research project has been approved by the Peter MacCallum Cancer Centre Human Research Ethics Committee and will be carried out in line with the National Statement on Ethical Conduct in Human Research (2007) – including all updates

Please complete the survey below.

Thank you!

Screening Question

Have you attended the Breast Mainstreaming in-service run by a member of the Parkville Familial Cancer Centre (PFCC)?	◯ Yes<br>◯ No<br>(Please select one option)

Part 1

All about you

Do you identify as:	◯ Female<br>◯ Male<br>◯ Other<br>◯ Prefer not to answer<br>(Please select one option)
What is your profession?	◯ Medical Oncologist<br>◯ Radiation Oncologist<br>◯ Surgeon<br>◯ Breast Care Nurse<br>◯ Fellow<br>◯ Other<br>(Please select one option)
Please describe your profession	________________________________
Years in current profession?	◯ 0-5 years<br>◯ 6-10 years<br>◯ 11-15 years<br>◯ 16-20 years<br>◯ More than 20 years<br>(Please select one option)
Where do you spend a majority of your professional time?	◯ Public<br>◯ Private<br>(Please select one option)
Which best describes the location of your practice?	◯ Metropolitan*<br>◯ Rural*<br>◯ Both
Apart from attending the Breast Mainstreaming in-service, have you had any formal training in cancer genetics?	◯ Yes<br>◯ No<br>(Please select one option)
Could you please provide details of what this cancer genetics training was?	________________________________

Part 2

This next section asks about your experience of the Breast Mainstreaming in-service

Do you feel that the in-service gave you the skills necessary to:

Discuss mainstream genetic testing with a patient?	◯ Yes<br>◯ No<br>◯ Unsure<br>(Please select one option)
Please explain why you feel the in-service didn't provide you with the skills to discuss mainstream genetic testing?	__________________________________
Consent a patient to mainstream genetic testing?	◯ Yes<br>◯ No<br>◯ Unsure<br>(Please select one option)
Please explain why you feel the in-service didn't provide you with the skills to consent a patient to mainstream genetic testing	__________________________________
Interpret a genetic test result for a patient?	◯ Yes<br>◯ No<br>◯ Unsure<br>(Please select one option)
Please explain why you feel the in-service didn't give you the skills to interpret a genetic test result	__________________________________

During the in-service:

	Strongly disagree	Disagree	Neither disagree or agree	Agree	Strongly agree
The objectives of mainstreaming were clearly defined	○	○	○	○	○
The content was relevant to my practice	○	○	○	○	○
The content was well organised	○	○	○	○	○
The content was easy to follow	○	○	○	○	○
The handouts were helpful to clarify the mainstreaming process	○	○	○	○	○
The time allotted was sufficient	○	○	○	○	○

Do have any suggestions for improvements that could be made to the in-service?

____
(Please comment)

Part 3

This next section asks about your personal experience of breast mainstream genetic testing in your clinic

Since attending the breast mainstreaming in-service, have any of your breast cancer patients been eligible* for mainstream genetic testing?	◯ Yes<br>◯ No<br>(Please answer yes, even if you have only seen patients who were eligible but you did not offer them mainstream genetic testing)
Have you raised the option of mainstream genetic testing with any eligible patients?	◯ Yes<br>◯ No
What are the reasons you haven't raised mainstream genetic testing with any eligible patients?	☐ Another member of the cancer treating team had this discussion with the patient<br>☐ Language barrier - patient did not speak English<br>☐ Patient required a more complex discussion at an FCC appointment<br>☐ I did not feel confident to discuss testing with the patient<br>☐ I did not have the time required to discuss with the patient<br>☐ Other<br>(Please select as many options that are relevant)
What other reasons do you have for not raising mainstream genetic testing with any eligible patients?	____<br>(Please comment)
Approximately how many patients have you raised the option of mainstream genetic testing with?	◯ 1<br>◯ 2<br>◯ 3<br>◯ 4<br>◯ 5<br>◯ 6<br>◯ 7<br>◯ 8<br>◯ 9<br>◯ 10<br>◯ 11<br>◯ 12<br>◯ 13<br>◯ 14<br>◯ 15<br>◯ 16<br>◯ 17<br>◯ 18<br>◯ 19<br>◯ 20<br>(Please select one option)
Have you seen any eligible patients who you have not offered mainstreaming to?	◯ Yes<br>◯ No

What are the reasons you haven't offered an eligible patient testing?	☐ Another member of the cancer treating team had this discussion with the patient<br>☐ Language barrier - patient did not speak English<br>☐ Patient required a more complex discussion at an FCC appointment<br>☐ I did not feel confident to discuss testing with the patient<br>☐ I did not have the time required to discuss with the patient<br>☐ Other<br>(Please select as many options that are relevant)
Did you refer these patients to local FCC?	◯ Yes<br>◯ No

During the in-service you were provided a document with a suggested script for initiating a conversation with a patient about mainstream genetic testing. Please consider this document when answering the following question.

	Never	Rarely	Sometimes	Always
Do you use the suggested script?	◯	◯	◯	◯

Could you please explain why you don't always use the script?

____
(Please comment)

In your clinic is there a medical specialist with cancer genetics experience that is allocated to see most or many of the breast cancer patients eligible for mainstream genetic testing?

◯ Yes
◯ No

Have you consented any breast cancer patients to have mainstream genetic testing?

◯ Yes
◯ No

What are the reasons you haven't consented any patients to have testing?

☐ Another member of the cancer treating team consented the patient
☐ Language barrier - patient did not speak English
☐ The patient required a more complex discussion at an FCC appointment
☐ Patient declined testing
☐ I did not feel confident to consent the patient
☐ I did not have the time to consent the patient
☐ Other
(Please select as many options that are relevant)

What other reasons do you have for not consenting any patients?

____
(Please comment)

Approximately how many patients have you consented for mainstream genetic testing?

◯ 1
◯ 2
◯ 3
◯ 4
◯ 5
◯ 6
◯ 7
◯ 8
◯ 9
◯ 10
◯ 11
◯ 12
◯ 13
◯ 14
◯ 15
◯ 16
◯ 17
◯ 18
◯ 19
◯ 20
(Please select one option)

	Very low	Low	Moderate	High	Very high
Please rate your confidence in consenting a patient to mainstream genetic testing	◯	◯	◯	◯	◯

What are the main factors that impact your confidence?

- ☐ My cancer genetics knowledge
- ☐ My ability to deliver pre-test counselling
- ☐ My ability to deliver the result post-test
- ☐ My understanding of the mainstreaming process
- ☐ The limited time I have to spend with each individual patient
- ☐ Other

(Please select as many options that are relevant)

Please describe what other factors impact your confidence to consent a patient for mainstreaming

____
(Please comment)

The next question relates to your personal experience consenting patients for mainstream genetic testing

When consenting a patient for mainstream genetic testing do you discuss the following:

	Never	Rarely	Sometimes	Always
How the sample will be collected	○	○	○	○
The cost* involved	○	○	○	○
That the results will only be used for clinical purposes and not research purposes	○	○	○	○
The mode of inheritance of hereditary breast cancer genes	○	○	○	○
Possible family implications	○	○	○	○
Privacy concerns	○	○	○	○
Possible insurance implications	○	○	○	○
The expected time* until results are available	○	○	○	○
The types of result expected (no mutation, mutation, variant unknown significance)	○	○	○	○
What the different types of results mean in terms of the risk of developing another cancer	○	○	○	○
What the different types of results may mean for current treatment plan	○	○	○	○

You have selected 'never' to one or more of the above questions. Could you please explain why you never discuss this aspect? __________________________________

When consenting a patient do you also:

	Never	Rarely	Sometimes	Always
Provide patient with a copy of the "Patient Information Sheet"	◯	◯	◯	◯
Discuss referring to FCC for further counselling/support	◯	◯	◯	◯

You have selected 'never' to one or more of the above questions. Could you please explain why you never undertake this aspect?

____
(Please comment)

After consenting a patient for mainstream genetic testing do you personally order* the test?

◯ Yes
◯ No
◯ Not personally because I am not eligible to sign the pathology slip
(Please select one option)

Who is likely to order the test?

____
(Please comment)

The next two questions refer to results disclosure for any patient that you have consented to mainstream genetic testing

	Never	Sometimes	Always
Do you deliver the result directly to your patients?	◯	◯	◯

A majority of the time who delivers the result to the patient?

◯ Another member of the cancer treating team
◯ The FCC directly deliver the result to the patient
◯ Other
(Please select one option)

Who delivers the result?

____
(Please comment)

Have any of your patients received a positive (mutation) result from mainstream genetic testing?

◯ Yes
◯ No
◯ Unknown

In most cases, did this change the patient's breast cancer treatment?

◯ Yes
◯ No
◯ Unsure
(Please comment)

The PFCC offer a phone number to contact a genetic counsellor to discuss any queries about the mainstreaming process.

Have you ever contacted the genetic counsellor on-call?	◯ Yes<br>◯ No<br>◯ I was unaware of this service<br>(Please select one option)

Part 4

This next section asks about your opinion of breast mainstream genetic testing

Question	Response
Do you believe that mainstream genetic testing should be part of your practice?	◯ Yes<br>◯ No
Why do you believe that mainstream genetic testing should not be part of your practice?	__________________<br>(Please comment)
Who do you believe should be responsible for facilitating mainstream genetic testing?	◯ Surgeon<br>◯ Oncologist<br>◯ Breast Care Nurse<br>◯ Fellow<br>◯ Genetic Counsellor embedded in a breast cancer clinic
Do you feel that you have been well supported by the Parkville Familial Cancer Centre during the implementation of mainstream genetic testing in your practice?	◯ Yes<br>◯ No
How could the PFCC better support you?	__________________<br>(Please comment)
What do you consider are some of the advantages of incorporating mainstream genetic testing in your practice?	☐ Streamlined process for your patients to access genetic testing<br>☐ Rapid turnaround time of genetic test results to inform patient's treatment decisions<br>☐ Working in direct partnership with PFCC to deliver the best care for your patient<br>☐ Other<br>☐ No advantages<br>(Please tick as many boxes that apply)
Please explain the other advantages of incorporating mainstream genetic testing in your practice?	__________________<br>(Please comment)
What do you consider are some of the disadvantages of incorporating mainstream genetic testing in your practice?	☐ Increased time involved to discuss all aspects of genetic testing with patient compared to standard care<br>☐ Increased management of patient's stress/anxiety raised by the nature of genetic testing and subsequent results<br>☐ Increased pressure placed on yourself to provide 'genetic counselling' for your patient<br>☐ The increased uncertainty for patient raised by genetic test results is not helpful for management of their current breast cancer diagnosis<br>☐ Other<br>☐ No disadvantages<br>(Please tick as many boxes that apply)

Please explain the other disadvantages of incorporating mainstream genetic testing in your practice?

____
(Please comment)

Can you think of any improvements to the mainstreaming process that would ensure the best care for your patients?

____
(Please comment)

Other comments

____
(Please comment)
